# Flirting With or Through Media: How the Communication Partners’ Ontological Class and Sexual Priming Affect Heterosexual Males’ Interest in Flirtatious Messages and Their Perception of the Source

**DOI:** 10.3389/fpsyg.2022.719008

**Published:** 2022-02-22

**Authors:** Jessica M. Szczuka

**Affiliations:** Social Psychology: Media and Communication, University of Duisburg-Essen, Duisburg, Germany

**Keywords:** digitalized sexuality, digitalized intimacy, voice assistants, computer-mediated communication, sexual arousal, human–computer interaction

## Abstract

Because technologies are frequently used for sexual gratification it seems plausible that artificial communication partners, such as voice assistants, could be used to fulfill sexual needs. While the idea of sexualized interaction with voice assistants has been portrayed in movies (e.g., “Her”), there is a lack of empirical research on the effect of the ontological class (human versus artificial) on the voice’s potential to evoke interest in a sexualized interaction and its perception in terms of sexual attractiveness. The Sexual Interaction Illusion Model (SIIM), which emphasizes influences on sensations evoked by artificial interaction partners, furthermore suggests that there may be contextual influences, especially sexual arousal, that may be crucial for the question of engaging in a sexualized interaction with an artificial entity. To empirically investigate whether the ontological class of the speaker (computer-mediated human in comparison to voice assistants) and the level of sexual arousal affects the heterosexual males’ interest in hearing more flirtatious messages and the perception of the communication partner’s sexual attractiveness, an online experiment with between subject design was conducted. Two hundred and fifty seven respondents were confronted with at least four, and voluntarily six messages from either a computer-mediated human or a flirtatious voice assistant, in interaction with being previously primed sexually or neutrally. The results demonstrated that the effect of sexual arousal was not prevailing on the interest in further messages and the attractiveness perception of the interaction partners, while the ontological class did so. Here, the voice assistant evoked more interest in further messages and the technology itself, while the computer mediated human was perceived to be more sexually attractive and flirtatious, and evoked more social presence. The communication partners social presence was shown to be the predictor with most explanatory power for the interaction partners perceived sexual attractiveness, regardless of whether it was human or artificial. The results underline differences between artificial and human interaction partners, but also underline that especially social presence and the feeling that the user is addressed (in terms of flirtatiousness) is crucial in digitalized intimacy regardless of the ontological class.

## Introduction

The voice plays an undeniably important role in sexual interactions. Besides the fact that human voice can be sexually arousing, it also provides cues important for partner perception and selection (e.g., sex, age, and health status, Babel et al., 2014). Research demonstrated that sexual auditory inputs can facilitate sexual arousal through a cognitive two-step process, consisting of an evaluation and a translation of the voice into a visual mental representation (Przybyla and Byrne, 1984). As artificial voices of machines become more human and sophisticated by using linguistic strategies that enhance authenticity (Google Duplex; Leviathan and Matias, 2018), market research surveys could already find that some people are aroused by their system’s voice or fantasize about their voice assistants and can even imagine falling in love with an Artificial Intelligence (AI) (e.g., Kaspersky, 2020). While commercial state-of-the art voice assistants are predominantly used to entertain (e.g., play music), control smart home features or gather knowledge among different domains (Ammari et al., 2019), the technology also has the potential to be used for ongoing conversations and represent a companion. Since throughout history, various technologies invented for a non-sexual purpose were then used for sexual gratification (e.g., telephone, photography, internet, Gordon, 1980), it stands to reason that voice assistants will meet the same fate. An example of what such sexual gratification with a voice assistant might look like has already been picked up in science fiction: In the movie *Her* (Jonze, 2013), a man develops a relationship with his voice assistant and starts to use the auditory input as a stimulus during masturbation.

The idea of people reacting toward media in the same way they would with other people is in line with the media equation theory, suggesting that media and therefore artificial entities can mindlessly activate social scripts, if equipped with social cues (Reeves and Nass, 1996). However, empirical research on digitalized sexuality is scarce and still aims to investigate whether mindless reactions toward media also apply to more intimate or sexualized interactions. The sexual interaction illusion model (SIIM) by Szczuka et al. (2019) conceptualized factors that can positively and negatively impact whether humans engage in sexualized interactions with artificial entities. One important concept, which is activated by the perception of a sexualized artificial stimuli (in the present study voice) is the sexual arousal that is evoked in the respondent. In line with research by Ariely and Loewenstein (2006) and Skakoon-Sparling et al. (2016), the authors argue that enhanced sexual arousal can be accompanied by an enhanced motivation to fulfill the aroused sexual need. This in turn could evoke a state comparable to a tunnel vision in which potential negative influences on the arousal (e.g., reflections on potential violations of sexual or social norms) are neglected. Following the SIIM, the evoked sexual arousal might then also interact with the evaluation of the interaction partner and the content of the sexualized interaction. Banks and van Ouytsel (2020) conducted an empirical study in which they explored reactions toward a sexualized chatbot compared to an interaction partner presented as a human. The results were mostly consistent with the SIIM, as they found that artificialness interfered with the arousal for some participants, while others engaged in the interaction because they had what the authors called a goodness-of-fit with either the content of the messages and/or the ontological class of the interaction partner. It is, however, questionable whether this also applies to synthetic voices and whether these have the potential to raise interest in further sexualized interactions.

The aim of the present study is to empirically investigate the effect of ontological class (human versus voice assistant) and sexual arousal on interest in the messages and the communication partner and its/her evaluation. Since previous studies (e.g., Szczuka and Krämer, 2017) as well as the SIIM suggest that engaging in digitalized sexuality may be affected by both, personal characteristics as well as evaluations of the technology/artificial persona. Both aspects are also investigated.

Sexual arousal and reactions toward potential flirtatious interactions strongly differ based on the person’s sex and sexual orientation. Moreover, males were found to have a more positive attitude toward online sexual activities (which thus includes voice assistants and computer-mediated messages; Shaughnessy et al., 2011). Therefore, this initial study focuses on processes within heterosexual males.

Research in this realm is not only important to understand more about human sexuality and how it is affected by technological advances, but also to gather data that may serve as a foundation for discussions about responsible handlings of technologies used within intimate interactions that have previously taken place between humans. The results provide further knowledge on the role of ontological class within sexual gratification and empirically tests assumptions of the SIIM.

## Theoretical Overview

### Communication, Sexuality, and the Role of Technologies

Acoustic signals of humans play an important role within mating and human sexuality (Levin, 2006). This includes vocal information of the person (e.g., the pitch, compare Leongómez et al., 2014), different vocalizations (especially during coitus), and the usage of language. Recent success of audio porn platforms (e.g., Dipsea, 2021; Femtasy, 2021; compare Cookney, 2020) demonstrated that there are numerous users getting sexually aroused by stories that feature erotic communication. The platforms promote that they evoke sexual fantasies, which are accompanied by sexual arousal. Researchers found that visual stimuli work as a moderator for the relationship between erotic auditive input and sexual arousal and that actual pictures of attractive persons are just as effective as mental imaginations (Przybyla and Byrne, 1984; Hawk et al., 2007).

As already addressed, technologies can play a role in mediating erotic communication, but also serve as interaction partners themselves. Consistent with Döring et al. (2021), technologies can be incorporated into sexualized interactions in three different ways; first, they can enable sexualized interactions by connecting people (e.g., online dating platforms). Second, technologies can mediate sexualized interactions (e.g., webcam sex), and finally, people can have a sexualized interaction with the technology itself (e.g., with sexualized chatbots or robots). The first two constellations focus on interpersonal contact between humans, which would include, for example, computer-mediated contact with another human (e.g., *via* chat). Messaging apps, often equipped with the option to call each other or leave voice messages, are particularly useful for spatially separated interaction partners by enabling a constant communication. Sexualized communicative interactions with technologies on the other hand include counterparts equipped with an artificial persona. These artificial interaction partners can have varying degrees of embodiment, ranging from text-only chatbots to voice-based voice assistants to graphic representations of virtual assistants and sex robots. All these formats have been used for sexual gratification. Given the work’s emphasis on voice and communication, voice assistants will be used as a research objective, since they are also frequently confronted with sexualized queries (UNESCO, 2019). While one may argue that some of the requests are meant to test the system, initial user studies demonstrated that people do have sexual fantasies about their voice assistant (Cherian and Pounder, 2017). As elaborated beforehand, the system can theoretically perform a sexualized interaction, similar to how humans interact with each other. Voice assistants already have the function of reading out messages that could theoretically be sexualized messages from another person. This leads to the question whether a sexualized voice assistant would be of interest to a human interaction partner. The basis for the assumption that people can engage in intimate interactions with artificial entities is the media equation theory, which assumes that people react toward technologies as they would if the interaction were with another human (Reeves and Nass, 1996). Szczuka et al. (2019) postulated the SIIM, which emphasizes the artificiality of the partner as a major influence on the willingness to engage in a sexualized interaction with an artificial partner. Sexual arousal, on the other hand, acts as a potential facilitator of the willingness to engage in an interaction that might differ from the interactions humans are used to with other humans. Therefore, the following sections will address sexual arousal and the type of interaction partner (computer-mediated or voice assistant) as potential inferences on the desire to start and continue a sexualized interaction with a human or an artificial partner.

### The Role of Sexual Arousal Within Sexualized Interactions

According to the SIIM, sexual arousal can play a crucial part in the process of facilitating a sexualized interaction with an artificial persona (Szczuka et al., 2019). Sexual arousal evokes physiological and psychological changes within the person, based on an existing or imagined stimulus and manifests the intention to engage in sexual behavior (Chivers, 2005). Skakoon-Sparling et al. (2016) conducted a study on the impact of sexual arousal on risk-taking and explained that the attentional focus shifts when humans are sexually aroused. They explain that the state is associated with a kind of “tunnel vision” that is focused on the self and gratification rather than on “distal factors” such as concern for others or future considerations” (p. 34). Consistent with this, Ariely and Loewenstein (2006) demonstrated that sexually aroused males rated different sexuality related activities (e.g., various bondage activities, threesome) as more attractive than when they were not aroused. Furthermore, ethical considerations of how to behave to obtain sexual gratification (e.g., “Would you encourage your date to drink to increase the chance that she would have sex with you?”, p. 94) become less important when aroused. The results underline the importance of intra-individual differences, or situational factors, in contrast to individual factors that may affect how people evaluate different sexuality related aspects. The SIIM aimed to translate this finding into digitalized sexuality and concluded that this may also mean that people who are sexually aroused do not question the artificialness of, for instance, the voice assistant and associated reflective thoughts that could suppress sexualized interaction (Szczuka et al., 2019). To test whether sexual arousal influences the perception of the interaction partners (both artificial and computer-mediated), the following hypothesis was formulated:

*(H1) Respondents who are sexually aroused and/or have sexual thoughts (a) have a higher interest in hearing flirtatious messages, (b) perceive the communication partner as more sexually attractive, (c) perceive the communication partner as more flirtatious, (d) have less interest in the technology than respondents who are not primed sexually.*

### Flirtatious Interactions and the Potential Effects of the Interactions Partner Ontological Class

The objective of the present study is to find out not only whether sexual arousal makes a difference in the partner perception and desire to hear more flirtatious messages, but also whether the ontological class of the interaction partner, here computer-mediated human or artificial voice assistant, makes a difference.

To better understand the objective of the study, it is helpful to define flirtatious interactions and potential influences on how these interactions are perceived. O’Farrell et al. (2003) defined flirting as communication which “expresses sexual interest, declaring the beginnings of sexual pursuit and demanding some sort of response” (p. 663). Henningsen et al. (2008) moreover noted that flirting can be understood as “a ubiquitous human activity” and further explained that “People may flirt in a wide array of settings and in a variety of different ways” (p. 483). In his research on motivations for flirting Henningsen (2004) found six different reasons for flirtatious communication. These include sexual motivation, relational motivation, fun motivation, exploring motivation, esteem motivation and instrumental motivation. Among other differences between how different people of different sexualities and sexes flirt, he demonstrated that females flirt significantly more often for fun reasons. This is in line with Guerrero et al. (2018) who stated that flirting is not necessarily related to a romantic relationship or sex. Researchers found that being the target of a flirt can be perceived as flattery and interest in the own person, which, consequently, can be associated with positive emotions and a confidence boost, even in a computer-mediated setting (Hall, 1993; Cialdini, 2009; Tanner and Tabo, 2018). It is likely that this boost of confidence is associated with the fact that flirting also represents a sign of genuine interest in another person (Hall et al., 2010).

There is a lack of research about whether flirting follows similar rules when the interaction partner is artificial. Especially because artificial social interaction partner until today do not develop their own motivations, potential differences in how males react to flirtatious messages of voice assistants could also be grounded in the missing ability to be sincerely motivated to flirt with the other. However, in line with the aforementioned media equation theory it is plausible that respondents interacting with an artificial entity will react toward it in a comparable manner as they would when interacting with another human (Reeves and Nass, 1996). At this point it needs to be mentioned that mindlessness is an important aspect of the theory. This implies that the reactions occur naturally because humans are fundamentally social, but that, reflecting on the interaction, respondents understand that the technologies do not warrant this kind of reactions. Consistent with this, research on sexualized technologies demonstrated how people evaluate sexual interactions with machines differently when asked explicitly and implicitly. Szczuka and Krämer (2017) demonstrated that heterosexual males do rate women to be more attractive than sexualized robots if asked explicitly through an survey, but that this difference disappears when affective priming is used to access evaluative strengths of attractiveness of both stimuli groups. Based on evolutionary psychological reasoning and familiarity, it is plausible that humans rate their own species as more appealing in comparison to artificial replications. However, social desirability and the potential to derive from social and sexual norms may also contribute to differences in perceptions of human and artificial sexual interaction partners. Moreover, media does portrait sexualized interactions with machines as something for people who experience difficulties in their social lives (Döring and Poeschl, 2019), and while this may be a valid and important point (compare Fosch-Villaronga and Poulsen, 2021), sexualized interactions with technologies have the potential to be appealing for a wide range of users (e.g., as they enable the user to act out sexual fantasies on his/her own). However, it should also be mentioned that research on the transmission of evolutionary psychological processes of mate perception demonstrated that heterosexual males have different gaze behaviors when confronted with a sexualized robot in comparison to a woman as they, for instance, paid more attention to the face, which is known to convey important emotional and motivational information (Szczuka and Krämer, 2019). And while this finding puts an emphasis on visual attention and sexualized robots, it can still be interpreted as a hint that, although the artificial stimuli evoke similar reactions in humans, authentic signals of partner perception could be unique within the own species as these signals are important not only for hedonistic (in terms of sexual gratification), but also evolutionary purposes (reproduction).

While no concrete research has yet been conducted on how voice assistants are perceived in this context compared to humans, Banks and van Ouytsel (2020) conducted an empirical study on how people interacted with a sexualized chatbot that either displayed human or machine-like cues (e.g., extended typing delays or visual and textual indicators of sex). While manipulation checks indicated that most of the respondents were aware that the chatbot showing human-like cues, was also a bot instead of a human, results also showed that the participants experienced sexual arousal across both conditions. Analysis of additional qualitative data demonstrated that some participants even climaxed based on the messages received from the chatbot. The authors discuss their findings by highlighting the importance of personal fit (as theoretically conceptualized within the SIIM, Szczuka et al., 2019) concluding that “it may be that the ontological class of the partner matters less than the sex-chat content and structure adherence to the norms and preferences for a fit partner” (p. 11). And although it should be interpreted carefully, as data was collected *via* self-report, a survey among voice assistant users demonstrated that almost one third of the respondents reported having sexual fantasies about their voice assistant (Cherian and Pounder, 2017; Kuzminykh et al., 2020).

In summary, there are empirical studies that provide reasons to believe that people evaluate and react to humans more positively compared to artificial entities in the context of digitalized sexuality (e.g., Szczuka and Krämer, 2017). However, there is also theoretical considerations (media equation theory, Reeves and Nass, 1996) as well as first qualitative reports that indicate that people may enjoy a sexualized interaction with an artificial entity (e.g., Banks and van Ouytsel, 2020). Based on these considerations, the following research questions were conducted:

*(RQ1) Is there a difference in (a) the interest to hear flirtatious messages, (b) the perception of the communication partner’s sexual attractiveness, (c) the perception of the interaction partner’s flirtatiousness based on the ontological class of the communication type (computer-mediated human vs. artificial voice assistant)?*

*(RQ2) Are there interaction effects that influence the perception of the communication partner based on the priming and the type of communication partner?*

### Personal Characteristics and Interaction Evaluations as Influences on Attractiveness Perceptions and Willingness to Engage in Sexualized Interactions

The willingness to engage in sexualized interactions with both artificial entities and humans is influenced by person characteristics but also by characteristics of the interaction partner, or technology.

Regarding personal characteristics, the study puts an emphasis on sociodemographic variables, attitudes revolving around human-computer-interaction and more sexuality-related characteristics. The sociodemographic variables that influence interest in sexualized interactions with an unknown person are age and relationship status. From an evolutionary psychological perspective, age plays an important role in mating and the way humans react to others who belong to the group of their preferred sex. Being or, in the case of the present study, sounding sexually mature can be a key component for females to attract heterosexual males at a wider range of age. This is grounded in women’s childbearing capacity at young to middle age. In line with this, research by Collins and Missing (2003) and Feinberg et al. (2008) shows a correlation between attractive voices and physical attractiveness, and that higher-frequency voices, an indicator of younger age, are rated as more attractive among male participants across a wider age range. Regarding voice assistants, a qualitative study by Kuzminykh et al. (2020) demonstrated that voices of commercial voice assistants, namely Apple Siri, Amazon Alexa, and Google Assistant, are perceived in the age range of 20–40 years, respectively middle aged, and therefore theoretically fertile women. Because both, the age of the recipient and the speaker as well as the voice itself and attractiveness ratings were shown to be connected, age was included as a covariate and predictor.

It is moreover likely that the relationship status as well as the sexual satisfaction of the participant plays a role in the willingness to engage in a sexualized interaction. While O’Farrell et al. (2003) demonstrated that singles and people in relationships differ in their responsiveness toward flirts, Hall et al. (2010) showed that flirting can also be platonic or intended to entertain the participants.

Regarding sexual satisfaction and relationship status as potential influences on willingness to engage in sexual interactions, research in pornography consumption provides a reason to include the variables. Even though pornography cannot be compared to sexualized communication, the idea that one may engage in it less because of the relationship status and/or sexual satisfaction has already been subject of research. While studies have found negative relations between sexual satisfaction and porn consumption, they also emphasize that it can “boost” one’s sex life (e.g., Dwulit and Rzymski, 2019; Miller et al., 2019). Because this is also plausible for sexualized interactions with a voice assistant, the variables were included in the present study.

The last two personality traits to be included are technology affinity and experiences people have had with sexual activities online. Both represent how open-minded and accepting people are toward technology, even in a sexualized context (Shaughnessy et al., 2011; Franke et al., 2019). A systematic literature review about motives for engaging in online sexual activities (including for instance pornography consumption and computer-mediated sexualized communication) demonstrated that the characteristics of the Internet itself are an important reasoning (Castro-Calvo et al., 2018). In accordance with the so-called triple A-Engine, the authors found that ease of access and associated safety, anonymity, and the ability to act out sexual fantasies, complemented by affordability (also in terms that sexual gratification can be achieved with less costs compared to offline) are important reasons why people engage in sexual online activities. A study by Daneback et al. (2012), however, found that private access to these internet resources seems to be crucial.

Despite these personality traits that might influence how people react toward digitalized sexuality, research on how people react and evaluate machines, as well as the SIIM, give reason to believe that the context, respectively the interaction and/or the technology used might influence how users respond. To understand whether the social perception of the persona, as well as technology related dangers might affect the evaluations, social presence and privacy concerns were included.

Social presence is defined as “feeling of being there with a “real person” (Oh et al., 2018, p. 1). The conceptualization of presence addresses the connection to the identity of an artificial interaction partner. Following Short et al. (1976) and Oh et al. (2018) discuss social presence in the realm of intimacy due to the connectedness that can be created during interactions in virtual settings. Regarding whether these aspects can play a role within digitalized sexuality it should be noted that the variable is close to what Szczuka et al. (2019) called sexual interaction illusion within the SIIM, a mental state in which the artificialness of the sexual interaction partner is not questioned. This state is crucial for the participation in a sexualized interaction with an artificial entity, as it (additionally to sexual arousal) shifts the focus on sexual gratification rather than potential deviations from sexual and social norms. Social presence has also been linked to perceived attractiveness (compare e.g., Fromberger et al., 2015; Croes et al., 2016) and was shown to be related to intention to meet a dating partner when meeting on a dating website (Jung et al., 2017). However, these studies have been primarily focusing on visual and informational stimuli (e.g., pictures, videos, information about geographical proximity) so far. Chérif and Lemoine (2019) conducted a study on the social presence of voices and found that human voices generate a stronger sense of social presence compared to more synthetic voices. This seems plausible against the background that social presence underlines the social and therefore human nature of an interaction partner. However, especially with better communication skills, this variable could play an important role in the investigation of digitalized sexuality with voice assistants.

The other important variable that could affect how people react toward sexualized online communication with a voice assistant or computer-mediated message is privacy concerns. Whenever technologies that are connected to the internet are used, it should be considered that data (from meta data about the user to data about the actual usage) can be stored. The EU’S General Data Protection Regulation (GDPR) has declared data about sexual orientation and sex life to be sensitive, and that it must only be processed if, for instance, the user gives explicit consent. However, there have been first incidents involving digitalized sexuality where sensitive data have been leaked or hacked (e.g., audio recordings derived from an app that simultaneously enabled control over a vibrator and sexualized communication or user data from online dating sites, Sundén, 2020). Moreover, it should be highlighted that different technologies are associated with different privacy concerns. Voorveld and Araujo (2020), for instance, demonstrated that interactions *via* smart speakers created higher data security concerns compared to the same interactions with a voice assistant on a smartphone. The authors discuss that this might be due to the fact that voice within the voice assistant is designed to continuously “listen” to users. Lau et al. (2018) argue that this could especially be due to missing transparency and complicated trust relations to the companies behind the state-of-the art speakers.

Based on the expounded importance of not only the personal characteristics but also the interaction evaluations, the following research questions and hypotheses were formulated:

*(RQ3) Are personal characteristics as well as the evaluations/perceptions of the communication (technology) predictors for (a) the interest to hear more flirtatious messages by a computer-mediated human, (b) the interest to hear more flirtatious messages by an artificial voice assistant, (c) the perceived sexual attractiveness of a computer-mediated human, and for (d) the perceived sexual attractiveness of an artificial voice assistant?*

*(H2a) The social presence within the sexualized messages with the human is higher in comparison to the artificial entity.*

*(H2b) The privacy concerns about sexualized communication and c) the interest in the technology is higher within interactions with an artificial voice assistant in comparison to the computer-mediated human.*

## Materials and Methods

In the following, the sample and procedure will be explained, followed details concerning the measurements. To meet the criteria of transparency and replicability, the used stimulus material and measures can be found online: https://osf.io/df79m/?view_only=356d50cbf6944ae8bf6382b0532bb33a.

### Sample

In total, 295 heterosexual male participants took part in the study. As the study had to be conducted online but contained elements which usually require assistance and attention (e.g., the priming and the thought experiment), quality fails, manipulation checks, as well as a long string analysis (Landers, 2020) were performed to ensure that the participants followed all instructions and actively participated in the study. The quality fails for instance included items checking for the participants careful response and the exclusion of participants who finished the questionnaire within a time that does not allow the participants to carefully read the instructions and participate in the study, while the long string analysis allowed for the detection of careless responders who, at the end of the questionnaire, had no variance in their response behavior (across multiple items/pages of items). Thus, 38 datasets were excluded from further analyses. Consequently, the data of 257 participants was used. To participate in the study, the respondents were asked to indicate whether they associated themselves to the male sex and whether they identified their sexuality as at least predominantly heterosexual in reference to the Kinsey Scale of Sexual Orientation (Kinsey et al., 1998). The age of the participants ranged from 20 to 85 (*M* = 43.74, *SD* = 16.74). Regarding the relationship status, 27.6% stated to be single while 72.4% indicated to be in a relationship or married. In line with the 2 × 2 design, four conditions that differed in priming and the communication partner were designed. The condition assignment was randomized. Eventually, 23.3% (*N* = 60) completed the condition including sexual priming and human message partner, 25.7% (*N* = 66) finished the sexual priming and voice assistant condition. Additional 26.1% (*N* = 67) took part in the neutral priming and human communication partner condition, whereas the remaining 24.9% (*N* = 64) participated in the neutral priming and voice assistant condition. The sample was recruited *via* two different panels to provide a more balanced sample of both, younger, as well as middle aged respondents.

The software program G*Power was used to conduct a power analysis. The goal of the present study was to obtain 0.95 power to detect a medium sized effect (*f*^2^ = 0.0625) at a standard 0.05 alpha error probability. The number of groups were 4, the number of predictors 2 and the response variables 6 (all these parameters were used to calculate the main effects for the priming and ontological class, and its interaction effects). The results of the *a priori* tests for MANOVA with special effects and interactions revealed a minimum sample size of 213 respondents. Following Dattalo (2013), G*Power can also be used to calculate the minimum sample size for a MANCOVA. This follow-up calculation showed that the sample should include at least 235 respondents.

### Procedure and Stimulus Material

After the participants had qualified for the present study (e.g., in terms of their sex, sexual orientation, and age) and completed the socio-demographic questions, they were confronted with questionnaires assessing the personal characteristics relevant for this study (affinity for technology, evaluation of the sex life and experiences in online sexual activities). The participants were then primed, either with sexual or neutral stimuli. To achieve an initial sexual arousal or a state in which the participants had neutral feelings, the participants were confronted with ten pictures, a video and the imagination task (which will be explained within this paragraph). To ensure that the used pictures and videos would induce either a state of sexual arousal or neutral feelings, both the photographs as well as the videos were pretested by 13 heterosexual adult males (the pretest also included pictures and videos for the neutral priming, which consequently were also pretested). Here, 50 pictures and 12 videos were rated regarding their level of eroticisms, how neutral they were perceived and the unpleasantness. Based on these ratings, ten neutral pictures, ten erotic pictures, three erotic scenes and three neutral videos (the three scenes were combined to one video) were chosen and used for the present study. As sexual arousal plays an important part in the present study, the priming needs to be explained in more depth. Even though other studies witch induced sexual arousal with pictures and videos showed sexually explicit behaviors and/or primary sexual characteristics (compare for instance Brand et al., 2011), this would have been problematic an online study because it was theoretically accessible to underaged persons. However, it is important to mention that the pictures showed erotic scenes, including naked women in sexualized poses, covering their primary sexual characteristics and couples during sexual activities covering their sexual characteristics with their poses. The video was composed of three different scenes which displayed sexualized content (women touching their bodies in underwear and a naked women and men laying on top of each other while the men is caressing the back of the female) which displayed women in underwear or naked without any visible primary or secondary sexual characteristics. No pictures included blurred parts or censor bars. Examples for the pictures used in the can be seen in the OSF project. The ten pictures used for the priming were presented for at least 50 s (five times repeated ten second timer in which two pictures were presented), whereas the video took about 20 s. In order to not only rely on the effects of the pictures and videos, the respondents were moreover instructed to take part in a thought experiment in which they were asked to think about a sexual fantasy they had (based on a method used by Birnbaum et al., 2012). In the neutral condition, the participants saw pictures and a video showing objects or landscapes (also pretested material). The respondents, too, participated in a thought experiment in which they were encouraged to think about their living room. The thought experiment was accompanied by a timer which gave the participants about 25 s to focus on their thoughts. To test whether the pictures, videos and thought experiment were successful, the participants indicated whether they felt sexually aroused, how strong their sexual thoughts were, how neutral the participants felt and how neutral their thoughts were. Four *t*-tests demonstrated that the priming worked within both groups. The participants confronted with the sexualized content had significantly stronger sexual thoughts [*M* = 3.86, *SD* = 1.33; *t*(234.63) = 14.61, *p* < 0.001, *Cohen’s d* = 1.83] than the respondents in the neutral condition (*M* = 1.69, *SD* = 1.02) and experienced a significantly higher sexual arousal [*M* = 3.33, *SD* = 1.39, *t*(241.79) = 10.78, *p* < 0.001, *Cohen’s d* = 1.35] compared to the participants who were confronted with neutral content (*M* = 1.61, *SD* = 1.14). In contrast to that, the participants in the neutral condition had stronger neutral thoughts [*M* = 4.63, *SD* = 1.37, *t(*249.54) = –6.917, *p* < 0.001, *Cohen’s d* = 0.87] than the participants in the sexualized condition (*M* = 3.37, *SD* = 1.53) and felt more neutral [*M* = 4.59, *SD* = 1.46, *t*(255) = –5.78, *p* < 0.001, Cohen’s *d* = 0.73] in comparison to the respondents in the sexual priming conditions (*M* = 3.52, *SD* = 1.47). This suggests that the participants were successfully primed.

**FIGURE 1:**
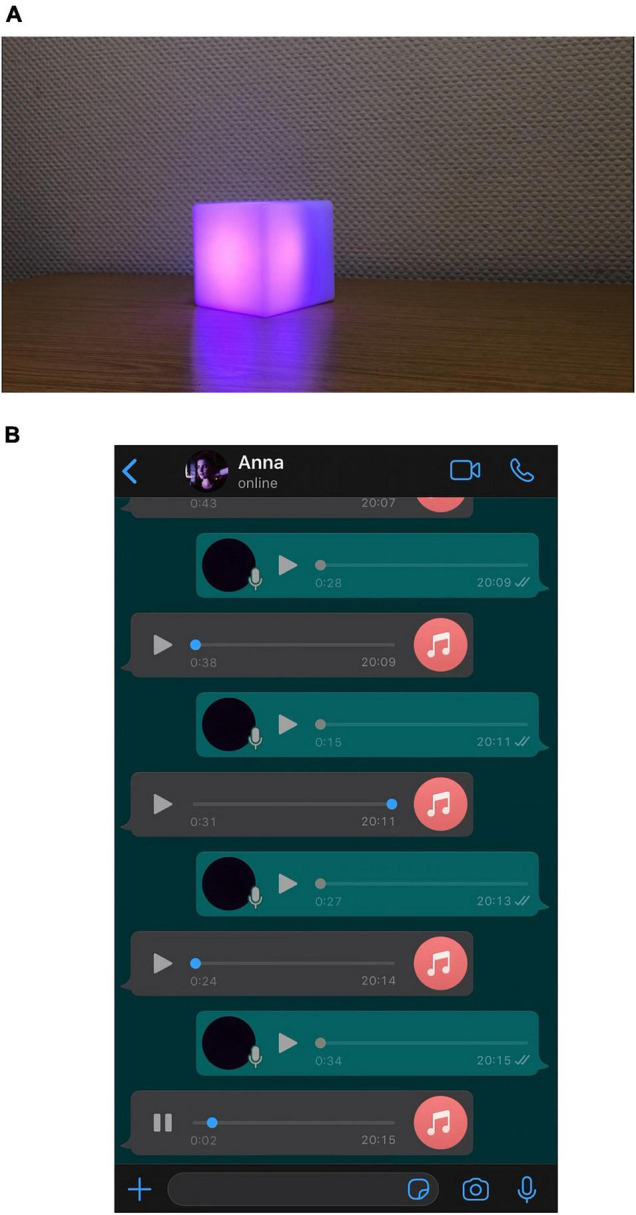
Stimulus material of the ontological class conditions. **(A)** Voice assistant. **(B)** Computer-mediated human.

After the priming, the participants were introduced to either the voice assistant named Ada, or the computer-mediated human called Anna. In both introductions, the participants were told that they would be presented with a new technology, a voice assistant that is focused on interpersonal and flirty interactions or a dating platform which focuses on voice messages between the humans as preferred communication form. The respondents then watched videos in which they could either see a cube lighting up during the spoken message (Figure 1A) or a messenger view in which voice messages could be heard (Figure 1B). Please note that the videos can also be found in the OSF project of the present study. Both conditions were spoken by the same female speaker and followed the same script. However, the conditions varied in the usage of human- and machine-like auditive cues. In the voice assistant condition there were, for instance, no sounds of breathing between the sentences (compare Wagner and Schramm-Klein, 2019), while the computer-mediated human voice messages contained different filler words (e.g., *I mean, like, uh, um*) which can be considered as social and personality markers (Laserna et al., 2014). The analysis of the item which asked to rate the partner in terms of human- and machine-likeness (a semantic differential that was rated on a five-point Likert scale ranging from 1 = *machine-like* to 5 = *human-like*) demonstrated that the conditions differed significantly in terms of the perceived human-likeness [*t*(253.33) = 11.19, *p* < 0.001, Cohen’s *d* = 1.40], with higher human-likeness ratings for the computer-mediated condition (*M* = 3.81, *SD* = 0.94) and a stronger tendency toward machine-likeness for the voice assistant (*M* = 2.42, *SD* = 1.04). In total, there were six videos, outputs or messages, of which only four were mandatory to watch and listen to. The last two videos were therefore optional. This choice was designed to quantify the participants’ interest in the messages. The videos had a length ranging from 21 to 35 s and aimed to cover subjects relevant in the initial contact and flirting phase. The first and second video focused on a person description and the expression of a desire to have a sexualized interaction online. The third and fourth video were composed of the description of a desired date (including what the speaker would wear and what they could do, implying that she would like to have a sexual interaction). In the optional fifth video, the protagonist asked how the interaction partner would imagine the date to be, including references to his sexual preferences. In the sixth and last optional video, the female protagonist highlighted how much she enjoyed the conversation, to a point where she feels sexually aroused and would therefore like to continue the conversation some other time. Each video was accompanied by the person perception measures described in the Measures section. Afterward, questions about the messages in general were asked. Lastly, the participants received an extensive debriefing.

### Measures

In the following, the used measures will be explained in more depth, separated by the perceptions and evaluations of the communication partners that are part of the main analysis (MANCOVA) and the personal traits that serve as additional predictors for further regression analyses.

#### Perceptions and Evaluations of the Communication Partner and Technology

##### Desire to Hear More Flirtatious Messages: Number of Messages/Outputs Heard by Choice

To investigate whether the participants were interested in hearing more flirtatious messages from the voice assistant or the computer-mediated human, they had the choice of whether to watch and listen to two additional messages of their communication partner (asked for one after the other). As there was no additional incentive for the participants to listen to the messages or outputs (e.g., monetary), it serves as a quantification of interest, intended to have a higher reliability than a theoretical self-report. The interest was coded into 1 (*no interest*), 2 (*low interest*) or 3 (*high interest*), depending on whether the participants wanted to listen to no, one or two additional messages. 37% (*N* = 47) of the participants who interacted with the computer-mediated human Anna wanted to hear no further message, 15.7% (*N* = 20) listened to one additional message and the majority of 47.2% (*N* = 60) wanted to hear all additional messages. The pattern was the same for the artificial communication partner Ada. Here, 37.7% (*N* = 49) had no interest in further outputs, followed by 13.1% (*N* = 17) who wanted to hear one additional output. Almost half of the participants who listened to the voice assistant (49.2%, *N* = 64) had a high interest in hearing two additional messages.

##### Perception of the Communication Partner’s Sexual Attractiveness

To measure the sexual attractiveness of the computer-mediated human and the voice assistant, different adjectives were rated with semantic differentials. In total, 13 adjectives were chosen from both the Godspeed Questionnaire (Bartneck et al., 2009) which aims to evaluate different aspects of how people perceive robots, and a measure used by study of Szczuka and Krämer (2017) which encompasses adjectives that have been used to measure attractiveness in the context of digitalized sexuality. Consequently, the items included different concepts ranging from attractiveness (e.g., sexually unattractive/sexually attractive) to anthropomorphism (e.g., machinelike/humanlike) to likeability (e.g., unfriendly/friendly). However, since I predominantly aimed to investigate the communication partner’s perceived attractiveness (with a focus on sexual attractiveness), four items related to the construct of sexual attractiveness were chosen. Consequently, the other nine items served as distractor items and should shift the focus from the perception of sexual attractiveness. The internal consistency of the used factor was α = 0.96. The mean of the computer-mediated human Anna was 3.36 (*SD* = 0.86), while the average perceived sexual attractiveness of the voice assistant Ada was lower with 2.56 (*SD* = 0.86).

##### Privacy Concerns

To measure whether the participants had privacy concerns with regard to the voice assistant Ada or the human computer-mediated communication partner Anna, three items of a scale by Xu et al. (2008) were adapted. Statements such as “*I am concerned that [the voice assistant/the human that was computer-mediated] is collecting too much personal information about me”* were rated on a five-point Likert scale ranging from 1 (*not at all*) to 5 (*very much*). The internal consistency of the factor was α = 0.91. The mean of the privacy concerns raised by the messages of the computer-mediated human was 3.59 (*SD* = 1.14) and for the interactions with the voice assistant 3.72 (*SD* = 1.18).

##### Social Presence

To measure whether the artificial and human communication partner evoked a sense of social presence, a subscale of a questionnaire that aims to evaluate virtual assistants by Gefen and Straub (2003) was used. The participants were asked to rate the five items (e.g., “*I felt a sense of human contact in [the voice assistant/the human that was computer-mediated]”*) on a five-point Likert scale ranging from 1 (*not at all*) to 5 (*very much*). The internal consistency was α = 0.96. The average social presence of the computer-mediated human (*M* = 3.23, *SD* = 1.15) was higher than of the artificial voice assistant (*M* = 2.26, *SD* = 1.14).

##### Interest in Technology or Content of Message

The interest in the used technology or the content of the sexualized messages was rated on two single items ranging from 1 *(uninteresting)* to 5 *(very interesting)*. For the computer-mediated human Anna, the mean for the interest in the used technology was 2.80 (*SD* = 1.42), whereas the average interest in the content of the messages was 3.24 (*SD* = 1.29). For the voice assistant Ada, the descriptive data revealed a mean of 3.00 (*SD* = 1.34) for the interest in the technology, and an interest in the content of the messages of 2.37 (*SD* = 1.24).

##### Flirtatiousness of the Communication Partner

Because attraction is facilitated by reciprocity, a single item was used to measure whether the participants felt that the communication partner was flirting with them. The item was measured on a five-point Likert scale ranging from 1 *(not at all)* to 5 *(very much)*. The mean of the item was higher for the messages of the computer-mediated human (*M* = 3.38, *SD* = 1.31) compared to the voice assistant (*M* = 2.44, *SD* = 1.22).

#### Personal Characteristics

##### Sociodemographic Variables

The two sociodemographic variables that were of interest were age and relationship status (single or relationship) which was assessed via self-report.

##### Affinity Toward Technology

To access whether the participants are drawn toward technology, the German version of the Affinity for Technology Interaction (ATI) Scale by Franke et al. (2019) was used. The scale consists of nine items, such as *“I enjoy spending time becoming acquainted with a new technical system”* that were assessed on a five-point Likert scale ranging from 1 (*not at all*) to 5 (*very much*) (*M* = 3.48, *SD* = 0.87). The internal consistency was α = 0.91.

##### Evaluation of the Own Sex Life: Sexual Satisfaction

Items from different scales were used to cover the multifaceted aspect of sex life, including the concepts of sexual self-esteem, sexual self-efficacy and the frequency of sexual encounters. The items were merged to add different perspectives to the evaluation of the own sex life and to encounter the problem that measuring all scales completely would have gone beyond the constraints of the online questionnaire’s length.

For the included items that captured whether participants have a positive appraisal of themselves in the context of sexuality, the Sexual Self-Esteem subscale from Ferrer-Urbina et al. (2019) was used. It was composed of four items such as “*I would not change anything in my current sex life.”* Moreover, an additional item from the Sexual Self-Efficacy subscale was included: “*I think I know how to stimulate my partner(s) well.*” Four items from Meston and Trapnell’s (2005) Sexual Satisfaction Scale were used to measure overall satisfaction with one’s sex life (e.g., *”I often feel that something is missing in my current sex life*”; reversed). Following Stulhofer et al. (2010), sexual satisfaction is associated with the frequency and variety of sexual encounters, which includes masturbation, sexual interactions with partners and sexual thoughts. Therefore, six items that were based on the New Sexual Satisfaction scale were included (e.g., “*Within the last six months, how satisfied have you been with the frequency of your sexual activities?*”). All items were answered on a five-point Likert scale.

To investigate the variance between the items, an explorative factor analysis using promax rotation was performed (Horn, 1965). The results yielded a two-factor solution with differences between items that asked for the evaluation of the sex life associated with other people and the rest (e.g., sexual solo-activities and sexual thoughts). Because the sexual satisfaction with not only the sexual encounters but more importantly also the self within these encounters is of more interest within the study the items were chosen that merely focused on the evaluations of the self. Therefore, the ten items with loadings higher than 0.500 were used for the present study. The factor measuring satisfaction with sexual encounters had a mean of 3.24 (*SD* = 1.13) and an internal consistency of α = 0.96.

##### Online Sexual Experiences

The Online Sexual Experience Questionnaire by Shaughnessy et al. (2011) was used to measure how experienced the participants were in terms of sexual gratifications that can be fulfilled online. The scale consists of ten items that cover activities associated with no arousal (e.g., “*Visited an educational web site on sexuality*”), solitary arousal (e.g., “*Masturbated while watching sexually explicit videos online*”) or partnered arousal (e.g., “*Repeatedly engaged in private discussion online about sexual fantasies with the same person*”). The items were rated on a five-point Likert scale ranging from 1 (*never*) to 5 (*very often*). The mean was 2.18 (*SD* = 0.78) and the internal consistency was α = 0.85.

## Results

### Effects of Sexual Priming and Communication Partner Conditions (H1a-d, RQ1a-c, H2a-c, RQ2)

A Multivariate Analysis of Variance and Covariance (MANCOVA) was computed to test whether both primary experimental conditions and the ontological class of the communication partner, affected the evaluations and perceptions of the communication partner. The dependent variables included were all evaluative measures about the messages and its sender. That is, the number of voluntarily heard additional messages or outputs (interest in further messages), the interaction partner’s sexual attractiveness, social presence and flirtatiousness, privacy concerns, and the interest in the technology. Because the literature suggests that the participant’s age and relationship status could influence how the interaction partner is perceived (see section on the influence of personal characteristics) but also whether the men want to listen to further flirtatious messages, both variables were included as covariates. Following Simon and Gagnon (1986), the sexual scripts may influence how assertive flirting communication is perceived. Therefore, the interest in the content of the message but also in the interaction partner (as it provides social cues that could be in favor of one’s scripts or not, e.g., the speaker’s voice that suggests her age) were furthermore included as covariates. The results of Box’s Test for Equivalence of Covariance Matrices and Levene’s Test for Equality of Variance demonstrated that the included variables meet the criterions of homogeneous covariance matrices [*F*(63,148082.72) = 1.02, *p* = 0.432] and homoscedasticity. The Kolmogorov–Smirnov test of normality revealed that the five dependent variables reached significance, except for the evaluation of the interaction partner’s sexual attractiveness. Meaning that they predominantly did not meet the criterion of normality. However, Following Field (2013), the used statistical methods (MANOVA and multivariate regressions) are both robust against this criterion, especially because the sample groups of the present study are large enough and equally distributed.

There was a significant main effect of the ontological class of the communication partner [*F*(6,244) = 10.483, *p* < 0.001; *Wilk’s*Λ = 0.795, $\eta_{\text{p}}^{2}$ = 0.205] on the combined dependent variables, but not of the priming condition [*F*(6,244) = 1.548, *p* = 0.163; *Wilk’s*Λ = 0.963, $\eta_{\text{p}}^{2}$ = 0.037]. Furthermore, no interaction effect between the ontological class of the interaction partner and the priming condition could be found [*F*(6,244) = 0.499, *p* = 0.808; *Wilk’s*Λ = 0.499, $\eta_{\text{p}}^{2}$ = 0.012]. After controlling for the participants’ age, relationship status, the interest in the content of the messages and in the interaction partner itself, the between-subjects effects demonstrated that the ontological class of the communication partner had an influence on the desire to hear more flirtatious messages [*F*(1,256) = 5.483, *p* = 0.020; $\eta_{\text{p}}^{2}$ = 0.022]. The descriptive data revealed that there was a slightly higher interest in hearing messages from the voice assistant (*M* = 2.11, *SD* = 0.92) compared to the computer-mediated human (*M* = 2.10, *SD* = 0.91). Moreover, the perception of the communication partner’s sexual attractiveness was significantly affected by the interaction partners ontological class [*F*(1,249) = 23.344, *p* < 0.001; $\eta_{\text{p}}^{2}$ = 0.086], with higher scores for the computer-mediated human Anna (*M* = 3.36, *SD* = 0.86) compared to voice assistant Ada (*M* = 2.56, *SD* = 0.87). Furthermore, the interaction partners significantly differed in terms of social presence [*F*(1,249) = 15.135, *p* < 0.001; $\eta_{\text{p}}^{2}$ = 0.057], with the computer-mediated human being perceived as more socially present (*M* = 3.23, *SD* = 1.15) than the voice assistant (*M* = 2.26, *SD* = 1.13). Another between-subjects effect of the type of interaction partner was shown regarding the perceived flirtatiousness [*F*(1,249) = 12.045, *p* < 0.001; $\eta_{\text{p}}^{2}$ = 0.046]. The computer-mediated human more strongly (*M* = 3.38, *SD* = 1.31) evoked the impression that she was flirting with the respondents than the artificial voice assistant (*M* = 2.43, *SD* = 1.22). The last significant effect of the communication partner condition was on the interest in the technology [*F*(1,249) = 23.855, *p* < 0.001; $\eta_{\text{p}}^{2}$ = 0.087]. The mean values demonstrate that the voice assistant evoked more interest in the technology (*M* = 3.00, *SD* = 1.33) compared to the computer-mediated human (*M* = 2.80, *SD* = 1.42). Consequently, the ontological class of the communication partner had no effect on the interaction-related privacy concerns [*F*(1,249) = 0.260, *p* = 0.611; $\eta_{\text{p}}^{2}$ = 0.001].

Even though the priming condition had no significant effect on the combined dependent variables, one significant between-subjects effect was found that should not remain unreported. The priming had an effect on the perceived flirtatiousness of the interaction partner [*F*(1,249) = 5.396, *p* = 0.021; $\eta_{\text{p}}^{2}$ = 0.021]. Respondents in the neutral condition (*M* = 3.12, *SD* = 1.34) felt more strongly that the interaction partner was flirting with them than the respondents in the sexualized condition (*M* = 2.66, *SD* = 1.32).

Taken together, almost all hypotheses concerning the sexual priming need to be rejected. The priming had either no effect (H1a, H1b, H1d, H1e) or unexpected effects in the neutral conditions (H1c; flirtatiousness of the communication partner). In contrast to this, the ontology of the communication partner affects almost all dependent variables. Consequently, RQ1a, RQ1b, and RQ1c can be positively answered as there are differences based on the ontological call of the communication partner. While the computer-mediated human Anna scored significantly higher on sexual attractiveness (RQ1b) and flirtatiousness (RQ1c), the voice assistant evoked more interest regarding additionally heard outputs (RQ1a). Since data revealed that the computer-mediated human evoked more social presence, H2a was supported. In terms of effects where the voice assistant scored higher than the computer-mediated human, H2b needs to be rejected as there were no differences regarding interaction related privacy concerns, while H2c can be accepted; the voice assistant evoked more interest in the technology. Because no interaction effects were found between the priming condition and the type of communication partner, RQ2 needs to be answered negatively.

### Characteristics of the Respondent and Communication Evaluations as Predictors of the Desire to Hear More Flirtatious Messages and the Perception of the Communication Partner’s Sexual Attractiveness

To investigate which personal characteristics and aspects of the communication with the computer-mediated human or artificial voice assistant predict the desire to listen to further messages and the perception of sexual attractiveness, four hierarchical linear regression analyses (two for each interaction partner) were conducted. The respondent’s characteristics age, relationship status, affinity for technology, sexual satisfaction, and experiences with online sexual activities were entered in the first block. The second block was composed of variables that evaluated the messages: the perception of the communication partner’s sexual attractiveness (which was only entered within the first two regressions as it served as a dependent variable for the latter two), the social presence of the communication partner, privacy concerns within the interaction, the feeling that the interaction partner was flirting with one, the interest in the used technology, and the interest in the content of the messages. The regression analyses were controlled for multicollinearity with a Variance Inflation Factor (VIF) below five (Menard, 2001). Please consider that answering the research questions required multiple analyses, which did not allow to control for the familywise error rate.

#### Predictors of Consuming Additional Flirtatious Messages (RQ3a and RQ3b)

As explained, the first two multiple regression analyses investigated the predictors of the additionally consumed messages. The hierarchical linear regression examining effects regarding the computer-mediated human revealed that the regression model which contained the respondent’s characteristics as predictors was non-significant [*F*(5,121) = 1.463, *p* = 0.207; *R*^2^ = 0.057, Adjusted *R*^2^ = 0.018], while the one containing the communication evaluation was significant [*F*(6,115) = 5.735, *p* < 0.001; *R*^2^ = 0.292, Adjusted *R*^2^ = 0.292]. The coefficient analysis revealed that, once the two blocks were entered, the only variable that predicts whether the participants chose to listen to additional messages by the computer-mediated human is the level of social presence she conveys [β = 0.376, *t*(126) = 2.57, *p* = 0.011]. It should, however, be mentioned that the experiences the respondents had had with online sexual activities were significant before the communication evaluations were entered in the second block. Table 1 presents all regression coefficients.

**TABLE 1:** Hierarchical regression analysis of additionally heard flirtatious messages of the computer-mediated human.

	Model 1			Model 2		
	*B* (*SE*)	β	*p*	*B* (*SE*)	β	*p*
Constant	0.85 (0.72)		0.244	0.02 (0.72)		0.977
Age	0.01 (0.01)	0.20	0.083	0.01 (0.01)	0.20	0.064
Relationship status	0.09 (0.20)	0.04	0.651	–0.03 (0.17)	–0.01	0.882
General sexual satisfaction	–0.01 (0.08)	–0.01	0.931	0.02 (0.07)	0.03	0.741
Experiences with online sexual activities	0.28 (0.12)	0.25	0.017	0.12 (0.10)	0.11	0.257
Affinity to technology	0.05 (0.10)	0.05	0.639	–0.08 (0.10)	–0.08	0.434
Perception of (sexual) attractivness				0.16 (0.15)	0.15	0.267
Social perception/presence				0.30 (0.12)	0.38	0.011
Perceived flirtatiousness of interaction				–0.10 (0.09)	–0.14	0.278
Interest in content of message				0.15 (0.09)	0.22	0.082
Interest in used technology				0.01 (0.07)	0.01	0.899
Privacy concerns				–0.04 (0.06)	–0.05	0.500
	*F*(5,121) = 1.46, *p* = 0.207, Adj. *R*^2^ = 0.02			*F*(11,115) = 5.74, *p* < 0.001, Adj. *R*^2^ = 0.29		

The same hierarchical linear regression was conducted to investigate the predictors of the number of additional messages heard from the voice assistant. The model for the first block, containing age, relationship status, affinity for technology, sexual satisfaction, and experiences with online sexual activities was only just significant [*F*(5,124) = 2.311, *p* = 0.048; *R*^2^ = 0.085, Adjusted *R*^2^ = 0.048]. The model composed of the communication evaluation variables (perception of Ada’s sexual attractiveness, her social presence, interaction related privacy concerns, the feeling that Ada was flirting with one, the interest in the used technology, and the content of the messages) was also significant [*F*(6,118) = 2.120, *p* = 0.024; *R*^2^ = 0.165, Adjusted *R*^2^ = 0.087]. All additional values are presented in Table 2.

**TABLE 2:** Hierarchical regression analysis of additionally heard flirtatious messages of the voice assistant.

	Model 1			Model 2		
	*B* (*SE*)	β	*p*	*B* (*SE*)	β	*p*
Constant	0.95 (0.64)		0.136	0.57 (0.71)		0.420
Age	0.01 (0.01)	0.11	0.286	0.01 (0.01)	0.14	0.154
Relationship status	0.03 (0.19)	0.01	0.878	0.13 (0.19)	0.06	0.493
General sexual satisfaction	–0.11 (0.08)	–0.14	0.140	–0.12 (0.08)	–0.14	0.141
Experiences with online sexual activities	0.29 (0.12)	0.24	0.017	0.23 (0.12)	0.19	0.061
Affinity to technology	0.18 (0.10)	0.15	0.085	0.14 (0.10)	0.12	0.175
Perception of (sexual) attractivness				0.03 (0.17)	0.03	0.852
Social perception/presence				0.13 (0.13)	0.16	0.313
Perceived flirtatiousness of interaction				0.05 (0.11)	0.06	0.672
Interest in content of message				0.06 (0.10)	0.08	0.548
Interest in used technology				–0.00 (0.07)	–0.00	0.992
Privacy concerns				–0.03 (0.07)	–0.03	0.708
	*F*(5,124) = 2.31, *p* = 0.048, Adj. *R*^2^ = 0.05			*F*(11,118) = 2.12, *p* = 0.024, Adj. *R*^2^ = 0.09		

The predictors formulated in RQ3a and RQ3b mostly need to be rejected. Even though the analyses showed significant regression models and some significant predictors, the explanatory power of the analyses is limited.

#### Predictors of the Communication Partner’s Sexual Attractiveness (RQ3c and RQ3d)

The next two hierarchical linear regressions were computed to investigate which variables predict the perception of Anna’s and Ada’s sexual attractiveness. As within the first two regressions, the first block contained personal characteristics (age, relationship status, affinity for technology, sexual satisfaction, and experiences with online sexual activities) while the second block was composed of communication evaluation variables (social perception, interaction related privacy concerns, the feeling that the interaction partner was flirting with one, the interest in the used technology, and the content of the messages).

The results of the analyses revealed that both the characteristics of the respondent [*F*(5,121) = 2.879, *p* = 0.017; *R*^2^ = 0.106, Adjusted *R*^2^ = 0.069] as well as the communication evaluations [*F*(5,116) = 27.195, *p* < 0.001; *R*^2^ = 0.701, Adjusted *R*^2^ = 0.675] explained significant variance in the perceived sexual attractiveness of the computer-mediated human Anna. The coefficients analysis demonstrated that the respondents’ age serves as the only personal characteristic that explains variance in the sexual attractiveness ratings of the computer-mediated human [β = 0.178, *t*(126) = 2.51, *p* = 0.014]. The jump in the explained variance between the models seems to be driven by the explanatory power of the perceived social presence [β = 0.544, *t*(126) = 6.37, *p* < 0.001] and the impression that the computer-mediated human was flirting with the respondent [β = 0.310, *t*(126) = 3.65, *p* < 0.001]. Table 3 presents all values.

**TABLE 3:** Hierarchical regression analysis of the computer-mediated humans perceived sexual attractiveness.

	Model 1			Model 2		
	*B* (*SE*)	β	*p*	*B* (*SE*)	β	*p*
Constant	2.37 (0.66)		<0.001	0.68(0.46)		0.137
Age	0.00 (0.01)	0.01	0.944	0.01 (0.00)	0.18	0.014
Relationship status	0.13 (0.18)	0.07	0.488	–0.06 (0.11)	–0.03	0.569
General sexual satisfaction	–0.05 (0.07)	–0.07	0.467	–0.04 (0.04)	–0.05	0.400
Experiences with online sexual activities	0.19 (0.11)	0.18	0.082	0.02 (0.07)	0.02	0.761
Affinity to technology	0.20 (0.09)	0.21	0.031	0.03 (0.06)	0.03	0.686
Social perception/presence				0.41 (0.06)	0.54	<0.001
Perceived flirtatiousness of interaction				0.20 (0.06)	0.31	<0.001
Interest in content of message				0.07 (0.06)	0.10	0.241
Interest in used technology				0.01 (0.04)	0.02	0.740
Privacy concerns				0.01 (0.04)	0.01	0.812
	*F*(5,121) = 2.88, *p* = 0.017, Adj. *R*^2^ = 0.07			*F*(10,116) = 27.20, *p* < 0.001, Adj. *R*^2^ = 0.68		

Another hierarchical linear regression with the same characteristics of the respondent and communication evaluations was computed to investigate which predictors explain variance in the perception of the voice assistant’s sexual attractiveness. The results demonstrated that the first block containing the personal characteristics was non-significant [*F*(5,124) = 2.074, *p* = 0.073; *R*^2^ = 0.077, Adjusted *R*^2^ = 0.040]. This was different for the communication evaluations with 67.7% explained variance [*F*(5,119) = 28.049, *p* < 0.001; *R*^2^ = 0.702, Adjusted *R*^2^ = 0.677]. The coefficient analysis revealed that the perceived sexual attractiveness of the voice assistant was significantly predicted by Ada’s social presence [β = 0.559, *t*(129) = 6.85, *p* < 0.001] and the feeling that the voice assistant was flirting with one [β = 0.294, *t*(129) = 3.76, *p* < 0.001]. Table 4 displays all other regression coefficients.

**TABLE 4:** Hierarchical regression analysis of the voice assistants perceived sexual attractiveness.

	Model 1			Model 2		
	*B* (*SE*)	β	*p*	*B* (*SE*)	β	*p*
Constant	1.99 (0.60)		0.001	0.79 (0.39)		0.043
Age	–0.00 (0.01)	–0.08	0.443	0.00 (0.00)	0.05	0.455
Relationship status	–0.32 (0.18)	–0.17	0.077	–0.08 (0.11)	–0.04	0.481
General sexual satisfaction	–0.03 (0.07)	–0.04	0.681	–0.08 (0.04)	–0.11	0.062
Experiences with online sexual activities	0.17 (0.11)	0.15	0.129	0.00 (0.07)	0.00	0.951
Affinity to technology	0.16 (0.10)	0.15	0.091	0.08 (0.06)	0.07	0.180
Social perception/presence				0.43 (0.06)	0.56	<0.001
Perceived flirtatiousness of interaction				0.21 (0.06)	0.29	<0.001
Interest in content of message				0.05 (0.06)	0.07	0.396
Interest in used technology				–0.00 (0.04)	–0.00	0.952
Privacy concerns				0.03 (0.04)	0.04	0.417
	*F*(5,124) = 2.07, *p* = 0.073, Adj. *R*^2^ = 0.04			*F*(10,119) = 28.05, *p* < 0.001, Adj. *R*^2^ = 0.68		

Even though the predictors formulated in RQ3c and RQ3d predominately need to be rejected, the large explanatory power of the regressions should be emphasized.

## Discussion

The aim of the present study was to investigate how sexual priming and the ontological class of a communication partner (artificial and computer-mediated) affects the willingness to engage in further messages, the attractiveness perception and other message evaluations. Within the following sections, the findings will be discussed.

### Effects of Sexual Priming (H1a-d, RQ2)

Literature suggests that sexual arousal might shift the focus on the aim to gain sexual satisfaction and therefore diminish thoughts about potential negative consequences or reflections on sexual norms (Ariely and Loewenstein, 2006). For this reason, potential differences in the reactions toward artificial and computer-mediated interaction partners based on sexual or neutral priming were investigated. Almost all hypotheses needed to be rejected. This is especially surprising as it would have been plausible that participants who were primed sexually also wanted to listen to more messages than those in the neutral condition to explore a potential increase in explicitness, which could have brought them even closer to sexual gratification. Even though participants in the sexual priming condition were significantly more aroused than those in the neutral condition (this is also underlined by large effect sizes) it is comprehensible that the shift of focus/attention to of instant sexual gratification needs a higher level of sexual arousal compared to the arousal evoked by showing sexualized images that (due to experimental considerations) could not be explicit. The experimental studies the hypotheses were based on had stronger manipulations of sexual arousal. Ariely and Loewenstein (2006) evoked sexual arousal by asking the participants to stimulate themselves (masturbate) before confronting them with questions, and Brand et al. (2011) facilitated sexual arousal with pornographic pictures that showed couples during intercourse or persons during masturbation. The missing interaction effects with the ontological class of the communication partner also suggest that the effect would not have worked with, for instance, the more familiar communication partner. In other words, sexual priming could have worked when followed by messages provided by a computer-mediated human in comparison to the voice assistant. This could have been in more accordance with the sexual script of the participants (Simon and Gagnon, 1986) which in the SIIM is one of the aspects summarized as goodness-of-artificial fit, which in turn is conceptualized to affect sexual arousal (Szczuka et al., 2019). Regarding the SIIM where sexual arousal is conceptualized as a driving force, results imply that sexual arousal needs to be redefined (in terms of how strong it needs to be) and empirically reinvestigated. The results of this study imply the need for higher levels of arousal.

### Effects of the Communication Partner’s Ontological Class (H1a-d, RQ2)

Theoretical considerations (especially in terms of the media equation theory, Reeves and Nass, 1996) suggested that there might not be any differences in the reactions people show regarding computer-mediated humans and artificial interaction partners, while first empirical results (e.g., Szczuka and Krämer, 2017; Banks and van Ouytsel, 2020) suggest differences based on the ontological class. In contrast to the effects of the sexual priming, the analyses suggest the ontological class to play an important role in the evaluation of sexualized messages. The results demonstrated that the computer-mediated human was evaluated to be significantly more sexually attractive and flirtatious, and that the human communication partner evoked more social presence compared to the voice assistant.

It is noticeable that especially being sexually attractive and flirtatious are attributes which, until now, have been used to describe human mating partners and seem not so easily transferable to artificial entities. Because the present study was conducted as a between-subjects design, interferences between the conditions are out of question. Regarding the sexual attractiveness, the findings is in line with other studies in which heterosexual men rated women to be more sexually attractive than artificial entities (here sexualized robots, Szczuka and Krämer, 2017).

In the realm of the SIIM (Szczuka et al., 2019) and in accordance with results of an empirical study by Banks and van Ouytsel (2020), the results (especially in terms of attractiveness and flirtatiousness) indicate that the voice assistant achieved no goodness-of-artificial fit, which might be due to constraints such as social and sexual norms (intimate or sexualized interactions are preserved for humans) but also evolutionarily rooted aversions (artificial humans may provide cues that deviate from healthy humans, e.g., voice, which might automatically trigger aversion). As the SIIM is based on the media equation theory, it also needs further research on whether it can be applied in the context of digitalized sexuality. Because the present study focused rather on evaluations than on observable reactions, a more profound understanding of the importance of mindless reactions toward technologies compared to humans is required.

The finding that the computer-mediated human was perceived as more flirtatious compared to the artificial communication partner tackles the importance of authentic cues within intimate interactions and the lack thereof within digitalized sexuality (discussed by Szczuka and Krämer, 2019). Among humans, flirting can fulfill personal needs (e.g., entertainment or boost of self-esteem) but also the aim to establish a sincere relationship between the involved (Hall et al., 2010). It is imaginable that the artificial interaction partner’s missing ability to build a deeper relation contributed to worse ratings in the flirtatiousness. However, the results of RQ3c and RQ3d both demonstrated that perceived flirtatiousness significantly explained variance in the sexual attractiveness ratings within the computer-mediated human and voice assistant. While one may argue that not every flirtatious communication between humans is driven by authentic social and sexual needs (compare telephone sex), implementing a convincing communication suggesting that machines have their own sexual needs and aims to engage in reciprocal communication may pose a difficult task for programmers. Further empirical research is needed to gain a deeper understanding of dyadic aspects of sexualized interactions within digitalized sexuality.

The difference in the social presence based on the communication partner’s ontological class is in line with Chérif and Lemoine (2019). This result is plausible as social presence per definition refers to the feeling of being connected with a “real” person (Oh et al., 2018). However, it can be of special interest for intimate interactions as social presence was found to be related to attractiveness (e.g., Fromberger et al., 2015). It is unclear whether the human voice is rated to elicit more attractiveness and social presence because both concepts find their origin in human nature or whether, contrary to this, the unnatural cues in the voice assistant condition broke the potential illusion of an interaction with a “real” person (also compare Szczuka et al., 2019). This result, however, highlights the importance of authenticity of voices in the context of digitalized sexuality, especially because social presence was a strong predictor for the interest in further flirtatious messages and the attractiveness ratings (see next section for discussion). In times of artificial voice systems which use numerous vocal cues to enhance human-likeness (e.g., Google Duplex), social presence could be improved in future technologies of digitalized sexuality.

It is questionable why the significantly higher ratings of attractiveness, flirtatiousness and social presence with the computer-mediated human were not accompanied by higher interest in further flirtatious messages. Instead, participants receiving messages from a voice assistant had significantly more interest in hearing further messages. It is likely that this effect was facilitated by the interest in the mere technology, which was also found to be significantly higher for the voice assistant. It seems plausible that the users wanted to get to know the system’s abilities to engage in a sexualized flirt, a style of communication which, until now, has mainly taken place among humans. Comparable effects in which users tested a system by confronting it with statements which, among humans, would cause emotional reactions (ranging from display of affection to insults) could be found for voice assistants (UNESCO, 2019) but also virtual agents (Kopp et al., 2005). However, it should be noted that the interest in the technology did not serve as a significant predictor for the interest in further messages of both communication partners. The result will be discussed in the following section.

Because research has demonstrated that voice assistants have a bad reputation concerning a lack of transparency on how data is processed and stored (Voorveld and Araujo, 2020), the missing effect of ontological class on the privacy concerns was surprising. However, the companies behind the state-of-the-art voice assistants are also frequently the reasons for mistrust (Lau et al., 2018), which was not the case within this study as the voice assistant was presented as a product of a university research project. The descriptive means show that the privacy concerns were only slightly higher for the participants confronted with the voice assistants, with the means of both ontological classes being over the scale average. It is further imaginable that participants had reservations to engage in an intimate interaction with an unknown person which, analog to the misusage of user data for the voice assistant, could misuse exchanged voice messages. More research is needed to understand the danger systems and companies, but also other users pose to privacy in the context of digitalized sexuality.

### Predictors of Consuming Additional Flirtatious Messages (RQ3a and RQ3b)

Another aim of the study was to investigate whether personal characteristics and communication evaluations would serve as predictors for the interest in further messages. The results indicate that for the voice assistant, the interest can clearly be attributed to the evaluation of the messages (explanatory power of about 30%) and, more precisely, to the social presence the sender evokes. Especially because the voice (and the person, respectively) was unknown (also without any further information one would normally get, e.g., on dating sites, such as age or hobbies), it seems plausible that the perceived connection to the computer-mediated human played an important role in the interest in further messages. This would be in line with Jung et al. (2017) who found that within dating sites, social presence (enhanced by more details within the dating profile) is connected with the willingness to meet the dating partner as it is stated to reduce uncertainty. This, however, is not the case for the voice assistant, as social presence did not significantly predict the interest in further messages. Future research should investigate whether cues that might facilitate social presence (e.g., a more human-like voice) also contribute to higher intentions to continue sexualized interactions with artificial entities. This would be in line with the SIIM which postulates that impressions of artificiality might negatively affect arousal and therefore the intention to engage in a sexualized interaction with an artificial entity (Szczuka et al., 2019).

The results concerning the predictors of interest in further messages from the voice assistant were less clear. Not only was the explanatory power of the regression models lower, but the coefficient analysis also revealed no significant predictors. One variable which still should be discussed as a variable which marginally reached level of significance is experiences with sexual online activities. Even though respondents in the voice assistant condition listened to more messages, the included variables do not seem to sufficiently depict the interest in these. This is especially interesting as interest in the technology was included in the calculation which would have been a plausible predictor considering people’s interest in testing artificial interaction partners in social interaction settings (e.g., Kopp et al., 2005). Not only does it reflect on the online experiences people have had with sexual activities but, on another level, how experienced people are with sexualized activities that include an unknown, physically absent counterpart (including the less interactive and personalized medium of pornography but also more interactive and personal forms like webcam sex, compare Shaughnessy et al., 2011; Castro-Calvo et al., 2018). It is therefore plausible that more experienced people in this format have less restrictions to engage in flirtatious messages sent by a computer-mediated human or a voice assistant. In line with the SIIM, this would support the idea that conflicting social and sexual norms can counteract sexual arousal in interactions with artificial entities as people who have engaged in sexual online activities may have a different mindset toward digitalized sexuality.

Intriguingly, the mere interest in and affinity for technology is not sufficient for intimate interactions with or through it. This brings up the discussion about the lack of predictive power for hearing more messages regarding attractiveness, perception of flirtatiousness and interest in the content of the messages. All of the above could have positively affected the arousal and therefore interest in the intention to continue a sexualized interaction according to the sexual script theory (Simon and Gagnon, 1986) and SIIM (Szczuka et al., 2019). It is imaginable that these variables are less important in a voice only setting compared to, for instance, graphical content. Here, users also had the chance to visualize an attractive person, which then could have contributed to the interest in further messages. This aspect was, however, not included in the present study and more research is needed to understand the importance of fantasy and uncertainty reduction in the context of digitalized sexuality. What was also striking was the missing effect of sexual satisfaction. This, though, is a comparable result to a study by Szczuka and Krämer (2017) who found that a person’s loneliness (which is likely to correlate with sexual dissatisfaction) does not automatically positively affect their attractiveness rating of sexualized robots. Needs of humans can therefore not automatically be addressed by digitalized sexuality. More importantly, there are other influencing factors (such as social presence) that explain people’s interest in a flirtatious interaction with or through media.

Lastly, privacy concerns did not function as a (negative) predictor either, which was especially likely in the voice assistant condition. As already discussed, claiming that the voice assistant was built for a university research project could have contributed to this as well as the fact that the participants were not asked to reply to the voice messages. More research is needed to understand the role of privacy concerns in digitalized sexuality.

#### Predictors of the Communication Partner’s Sexual Attractiveness (RQ3c and RQ3d)

In contrast to the interest to hear more messages, the results of the regression concerning the communication partner’s perceived sexual attractiveness showed a more consistent pattern. The models containing the personal characteristics and the evaluations of the communication partner explained 67% of the variance in the attractiveness ratings for both, the computer-mediated human and the voice assistant. In both cases, the coefficient analysis revealed that social presence was the most important significant predictor, followed by the impression that the communication partner was flirting with one. The result demonstrating that the perception of flirtatiousness also explains variance in both cases points out that the illusion of reciprocal exchange and the users feeling of “being addressed” may play an important role for digitalized sexuality, no matter if the impression is conveyed by a computer-mediated human or an artificial entity. Because flirtatious communication usually occurs among humans who are interested in each other, it is plausible that, comparable to this, respondents perceived the communication partner as more attractive if the feeling was “mutual,” i.e., if the respondents also served as an “object of desire.” This underlines that artificial interaction partners not only need to convey the illusion of own sexual needs but also need to incorporate dyadic interactions that allow the users to feel addressed. It is interesting that these social aspects, which are based on social behaviors among humans, served as predictors for sexual attractiveness instead of, e.g., the interest in the content of the messages, which underlines the importance of a social connection between the user and the interaction partner. The result moreover implies that the predictors which explain attractiveness ratings between humans can be transferred to artificial communication partner, even though the computer-mediated human was perceived to be significantly more attractive than the voice assistant.

The only difference in the regression analyses was that age served as an additional, positive predictor for the attractiveness evaluation of the computer-mediated human. Older participants might have felt flattered by the computer-mediated human who was spoken by a female who was younger than the mean participant, which resulted in higher attractiveness ratings (compare Hall, 1993). The importance of age is rooted in an evolutionary psychological mechanism which represents the other explanation. Feinberg et al. (2008) demonstrated that high pitched voices, which is an indicator for younger age and fertility, were related to attractiveness ratings. The role of authentic cues and evolutionary psychologically rooted mating mechanisms in digitalized sexuality was already discussed in a work by Szczuka and Krämer (2019), and since age did not serve as a predictor for the attractiveness ratings for the voice assistant, this should be emphasized in future studies.

### Limitations and Future Research

Because of the coronavirus pandemic, the study needed to be conducted online which came with two bigger limitations. First, future studies should rely on sexually explicit material to elicit sexual arousal. Especially because internet pornography had a huge impact on how people assess sexually explicit content, studies that aim to investigate the effects of sexual arousal should rely on comparable stimulus material shown to evoke sexual arousal (e.g., Brand et al., 2011). Secondly, it would be fruitful to investigate how participants reacted if confronted with the actual devices of the voice assistant or the computer-mediated human. In this case, it would have also been possible to investigate whether and if, how participants would have replied.

Especially because empirical research on digitalized sexuality is scarce (compare, e.g., Döring et al., 2020, on sexualized robots), the present study aims to inspire future research questions. The results underlining the importance of social presence and the experiences with online sexual activities shed a light on variables in the context of digitalized sexuality which should be included in future studies. Because social presence was higher for the computer-mediated human and connected to both, interest in further messages and attractiveness ratings, the study provides further evidence that human-like cues could be important in digitalized sexuality. However, too much human-likeness can also be harmful for the perception of artificial entities (compare uncanny valley theory). More research is needed to understand which cues can positively affect the perception of artificial entities in sexualized interactions.

Moreover, the study is the first of its kind to center on voice in the context of digitalized sexuality. Because auditive signals can facilitate sexual fantasies it would be interesting to gain more knowledge about how artificial voices may trigger processes of imagination in which the communication partner is artificial or human. This may also be helpful to get a better understanding of whether sexualized interactions with artificial entities are accepted (Compare SIIM; Szczuka et al., 2019) or whether people rather imagine interactions with interaction partners they are familiar with and which belong to their own species.

It also needs to be mentioned that the included dependent variables predominantly failed to reach the criterion of normality. As already mentioned within the results section, literature (Field, 2013) suggests that the used analyses methods are robust against violations of the criterion of normality, especially because the used sample groups are equally distributed and large enough. It, however, needs to be kept in mind when discussing the results. The fact that the evaluation of the non-human and human speakers’ sexual attractiveness was normally distributed is in favor of consistent data. However, the analyses of the histograms demonstrated that variables such as privacy concerns and the evaluated flirtatiousness of the source showed clear tendencies to one side. As in every study in the field of digitalized sexuality it needs to be questioned, whether there might be ceiling effects of social desirability. However, more research is needed to understand whether this is an effect associated with the topic of sexualized messages that are perceived from technological devices.

Lastly, the study raised numerous questions that could be addressed in follow up studies. First and foremost, there are different sample groups that should be considered in future studies, including humans of different gender and sexuality. Especially because erotic audio stimuli seem to be particularly interesting for female users (compare e.g., Cookney, 2020; Dipsea, 2021; Femtasy, 2021) while empirical research found no consent to how female and male differ in their reactions toward auditive erotic content (compare Chivers et al., 2010) more research (also with an emphasis on the stimuli’s ontological class) can help getting a better understanding on gender specific connections between cognitive mechanisms and arousal, in combination with the question of potential differences in reactions to artificial stimuli.

## Conclusion

With the rise of possibilities to interact with artificial entities *via* voice, the question arises whether this technology might also be used for sexual gratification. The goal was to get a better understanding of whether humans can be drawn to voice-based artificial entities and of what importance human cues are in voice-based digitalized sexuality. Therefore, this study investigated whether the perception of a communication partners sexual attractiveness and the interest to hear more flirtatious messages are affected by the ontological class of the communication partner (computer-mediated human vs. voice assistant) and whether these effects are influenced by the respondent’s sexual arousal. Within the present setting, sexual arousal was not prevailing while the communication partner’s ontological class had numerous effects. The computer-mediated human evoked higher levels of sexual attractiveness, social presence, and flirtatiousness, while the voice assistant evoked more interest in the technology and the desire to hear more messages. Taken together, the results introduce social presence and the importance of dyadic interest (perceived flirtatiousness) as important to the context of digitalized sexuality. Referencing this paper’s title, data demonstrates that flirting with another human *through* media allows one to form a stronger social connection to the communication partner (in terms of presence and flirtatiousness) which again underlines that humans, in comparison to artificial entities, represent a gold standard of sexuality.

## Data Availability Statement

The datasets presented in this study can be found in online repositories. The names of the repository/repositories and accession number(s) can be found below: https://osf.io/df79m/?view_only=356d50cbf6944ae8bf6382b0532bb33a.

## Ethics Statement

The studies involving human participants were reviewed and approved by Ethics Committee of the Division of Computer Science and Applied Cognitive Sciences at the Faculty of Engineering of the University of Duisburg-Essen. The participants provided their written informed consent to participate in this study.

## Author Contributions

The author confirms being the sole contributor of this work and has approved it for publication.

## Conflict of Interest

The author declares that the research was conducted in the absence of any commercial or financial relationships that could be construed as a potential conflict of interest.

## Publisher’s Note

All claims expressed in this article are solely those of the authors and do not necessarily represent those of their affiliated organizations, or those of the publisher, the editors and the reviewers. Any product that may be evaluated in this article, or claim that may be made by its manufacturer, is not guaranteed or endorsed by the publisher.
